# An Adaptive Deep Learning Optimization Method Based on Radius of Curvature

**DOI:** 10.1155/2021/9882068

**Published:** 2021-11-10

**Authors:** Jiahui Zhang, Xinhao Yang, Ke Zhang, Chenrui Wen

**Affiliations:** School of Mechanical and Electrical Engineering, Soochow University, Suzhou 215006, China

## Abstract

An adaptive clamping method (SGD-MS) based on the radius of curvature is designed to alleviate the local optimal oscillation problem in deep neural network, which combines the radius of curvature of the objective function and the gradient descent of the optimizer. The radius of curvature is considered as the threshold to separate the momentum term or the future gradient moving average term adaptively. In addition, on this basis, we propose an accelerated version (SGD-MA), which further improves the convergence speed by using the method of aggregated momentum. Experimental results on several datasets show that the proposed methods effectively alleviate the local optimal oscillation problem and greatly improve the convergence speed and accuracy. A novel parameter updating algorithm is also provided in this paper for deep neural network.

## 1. Introduction

Deep neural network has made great achievements in the field of computer vision, such as face recognition [1] and object detection [2]; by deepening the network depth and enriching the datasets constantly, deep neural network has significantly improved the recognition accuracy. However, the improvement of the network framework and the continuous enrichment of the datasets have certain limits for the improvement of the performance of the image classification task, which not only costs a lot of computing costs but also has great difficulty in general applicability and operation. As a key part of neural network training, the optimizer defines the method of updating the parameters of the depth model, which produces different effects in the training process. Compared with other improved methods, the improved optimizer has the advantages of simple replacement and wide application range. According to the learning rate, optimizers can be divided into two categories: one is a manually adjusted learning rate optimizer, such as SGD [3], SGD-M [4], and NAG [4], and the other is an automatically adjusted learning rate optimizer, such as AdaGrad [5], RMSProp [6], and Adam [7]. The auto-tuning learning rate optimizer automatically adjusts a separate learning rate for each parameter so that training can converge faster. However, Wilson et al. [8] found that the auto-tuning optimizer might converge to different local minima [9] due to its poor generalization effect. Otherwise, the experimental results of the auto-adjusting learning rate optimizer [10, 11] are ultimately difficult to match the manually adjusting learning rate optimizer. Therefore, the manual adjustment is targeted to improve the learning rate optimizer. SGD-M, as a widely used manual-tuning optimizer, would get better performance in many deep neural networks.

SGD-M updates network parameters by taking into account both past and present gradients. However, SGD-M has the problem of local optimal oscillation [12], which hinders the progress of gradient decline and requires more training time and training data. In addition, the instability of the model at the initial stage of training brings great oscillation to the system. SGD-M has different problems at different stages of gradient descent, so it is of great significance to design a new optimizer that can maintain good precision and converge faster. An adaptive clamping optimization algorithm (SGD-MS) is proposed in this paper based on the curvature radius, which adds the moving average term of future gradient on the basis of SGD-M. The thresholds are set to adaptively separate the momentum term and the future gradient cumulative term by considering the internal relationship between the curvature radius of the objective function and the gradient descent of the optimizer [13]. Three switching modes are introduced in our SGD-MS, including *V* (velocity) model with momentum term only, *D* (difference) mode with the future gradient moving average term only, and *S* (sum) mode with both terms, which effectively alleviates the problem of local optimal oscillation caused by the accumulation of momentum term and the instability of the system caused by the large future gradient at the beginning of training. In addition, SGD-MA, an accelerated version of SGD-MS, is designed based on the aggregated momentum [14] to realize adaptive change of the damping coefficient, which greatly accelerates the convergence rate.

The main contributions of this paper are as follows. (1) It is effective to alleviate the problem of instability in the initial stage by adding the term of future gradient moving average. (2) An adaptive switching method based on radius of curvature is proposed. The proposed SGD-MS algorithm will switch adaptively in three modes to adapt to different training stages and effectively alleviate the problem of optimal oscillation. (3) An accelerated version SGD-MA is proposed with the adaptive damping coefficient, which further accelerates the convergence speed. (4) The method in this paper is systematically evaluated on PASCAL VOC, CIFAR10, CIFAR100, and MNIST miniature datasets, which demonstrates the effectiveness of our proposed optimizer.

## 2. Related Work

The following introduces several typical principles and improvement methods of the manual adjustment learning rate optimizer.

SGD optimizer is the most basic algorithm for deep neural network optimization, whose update rule is shown as follows:

(1) $$\begin{array}{c} \theta_{t+1}=\theta_{t}-\frac{r\;\partial L_{t}}{\partial \theta_{t}}, \end{array}$$

where *t* is the current update step, *θ* represents the model parameters to update, and ∂*L*_*t*_/∂*θ*_*t*_ means the back propagation gradient of *θ*. The parameter *r* represents the learning rate of SGD, which determines the size of the updates in the current step.

However, SGD update direction is completely dependent on the current batch, which makes the update unstable. The SGD-M optimizer adds a momentum term on the basis of the SGD optimizer. The momentum algorithm accumulates the moving average of the past gradient with exponential attenuation, which makes the direction of the movement consistent. The update rules for the SGD-M optimizer are proposed in

(2) $$\begin{array}{c} \left\{\begin{array}{c} V_{t+1}=\alpha V_{t}+\frac{\partial L_{t}}{\partial \theta_{t}},\\ \theta_{t+1}=\theta_{t}-rV_{t+1}, \end{array}\right. \end{array}$$

where the momentum term *V* represents the moving average attenuation over the past gradient. The parameter *α* is the moving average decay rate, which is generally set at 0.9. Formula (2) is rewritten as follows:

(3) $$\begin{array}{c} \theta_{t+1}=\theta_{t}-\frac{r\;\partial L_{t}}{\partial \theta_{t}}-r\left(\sum_{i=0}^{t-1}\left(\frac{\partial L_{i}}{\partial \theta_{i}\alpha^{t-i}}\right)\right). \end{array}$$

It is evident that the parameters in formula (3) are updated based on the current gradient *r*  ∂*L*_*t*_/∂*θ*_*t*_ and the cumulative of past gradients  *r*(∑_*i*=0_^*t*−1^(∂*L*_*i*_/∂*θ*_*i*_*α*^*t*−*i*^)).

The NAG optimizer is a variant of momentum algorithm driven by the Nesterov acceleration gradient method [13]. The update rules of the NAG optimizer are given by the following formula:

(4) $$\begin{array}{c} \left\{\begin{array}{c} V_{t+1}=\alpha V_{t}+\frac{\partial L_{t}}{\partial \left(\theta_{t}+\alpha rV_{t}\right)},\\ \theta_{t+1}=\theta_{t}-rV_{t+1}. \end{array}\right. \end{array}$$

The following results are obtained by substituting ${\hat{\theta }}_{t}=\theta_{t}+\alpha rV_{t}$ into formula (4):

(5) $$\begin{array}{c} \left\{\begin{array}{c} V_{t+1}=\alpha V_{t}+\frac{\partial L_{t}}{\partial {\hat{\theta }}_{t}},\\ {\hat{\theta }}_{t+1}={\hat{\theta }}_{t}-\alpha^{2}rV_{t}-\frac{\left(1+\alpha \right)r\;\partial L_{t}}{\partial {\hat{\theta }}_{t}}. \end{array}\right. \end{array}$$

From the above formula, it is concluded that the NAG optimizer adds a correction factor to the momentum term on the basis of the SGD-M optimizer, which advances half a step forward in the process of parameter updating to achieve faster convergence. However, in the case of stochastic gradient, Nesterov's momentum has little effect on the convergence rate. Besides, it is unable for the algorithm to update different parameters according to their importance.

In order to solve the overshoot problem in the SGD-M optimizer and the NAG optimizer, the PID optimizer [15–17] imitates the principle of the PID controller to add a differential term in SGD-M. The update rules of the PID optimizer are as follows:

(6) $$\begin{array}{c} \left\{\begin{array}{c} V_{t+1}=\alpha V_{t}+\frac{\partial L_{t}}{\partial \theta_{t}},\\ D_{t+1}=\alpha D_{t}+\left(1-\alpha \right)\left(\frac{\partial L_{t}}{\partial \theta_{t}}-\frac{\partial L_{t-1}}{\partial \theta_{t-1}}\right),\\ \theta_{t+1}=\theta_{t}-rK_{i}V_{t+1}-rK_{d}D_{t+1}, \end{array}\right. \end{array}$$

where *V* and *D* are regarded as the integral term and the differential term of the PID controller, respectively. *K*_*i*_ and *K*_*d*_ are the adjustment coefficients for the integral term and the differential term, which is similar to the PID adjustment method and needs to be adjusted manually in the experiment. The design structure of the PID algorithm provides inspiration for our optimizer.

## 3. The Design of the Improved Optimizer

Since only the past and present information are used in the SGD-M optimizer during parameter update, a derivative term is added in the SGD-M optimizer to introduce future information transformation. The training of the deep model is usually based on small batch, which may introduce noise in gradient calculation. The moving average term *D* of future gradient part is proposed to alleviate this problem. The rules of the parameter update in (*t* + 1) iterations are shown as follows:

(7) $$\begin{array}{c} \left\{\begin{array}{c} V_{t+1}=\alpha V_{t}+\frac{\partial L_{t}}{\partial \theta_{t}},\\ D_{t+1}=\alpha D_{t}+\left(1-\alpha \right)\left(\frac{\partial L_{t}}{\partial \theta_{t}}-\frac{\partial L_{t-1}}{\partial \theta_{t-1}}\right),\\ \theta_{t+1}=\theta_{t}-rV_{t+1}-rD_{t+1}. \end{array}\right. \end{array}$$

It is seen from equation (7) that the SGD-M optimizer with *D* term is very similar to the PID optimizer. The difference is that the PID optimizer introduces super parameters *K*_*i*_ and *K*_*d*_. Although *K*_*d*_ is initialized by using Laplace transform theory and Ziegler Nichols tuning method, it still needs to be adjusted manually to achieve good results. Moreover, the objective function obtains the same convergence rate in different directions with fixed moving average decay rate *α*, which hinders the gradient descent process. The internal relationship between the curvature radius and optimizer gradient descent will effectively solve this problem.

### 3.1. The Relationship between the Radius of Curvature and the Gradient Descent

In mathematics, curvature usually indicates the curvature degree of a curve at a certain point. The radius of curvature is usually expressed as the reciprocal of curvature, which is used to describe the curvature degree of a curve at a certain point. Therefore, it is considered that the curvature at each point of the uneven curve is usually different from each other. The mathematical formula of the radius of curvature is written as follows:

(8) $$\begin{array}{c} K=\left|\frac{\hat{\nabla }L}{{\left(1+\nabla L^{2}\right)}^{3/2}}\right|,\\ \rho =\frac{1}{K}, \end{array}$$

where $\hat{\nabla }L$ is the second derivative, ∇*L* is the first derivative, and *ρ* is the radius of curvature.

**Figure 1:**
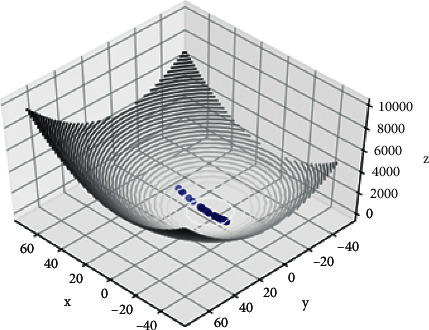
Iteration path of SGD-M algorithm.

It is shown the linear convergence of curvature changes robustness in SGD-M, which means the linear convergence of nonconvex objects with variable curvature. In addition, curvature is the only feature that describes the convergence of gradient descent [18]. Based on this feature, the proposed SGD-MS optimizer takes the radius of curvature as the evaluation criterion for the degree of convergence.  Figure 1 shows the result of 20 iterations for the SGD-M optimizer to find the minimum value of the Rosenbrock function [19]. The initial point is set as (1,0). According to equation (8), it is seen from the three-dimensional figure that the closer the gradient descent is to convergence, the smaller the mean curvature radius is. With this in mind, our algorithm takes the radius of curvature of the objective function as the threshold to switch modes according to the degree of convergence.

In the deep learning network, the parameter space has a more complex structure than the vector of Euclidean space. The parameters constitute a *m∗n* matrix, where *m* is the number of features and *n* is the number of classes. The number of input nodes in the neural network is the sum of input nodes in each layer. The parameter space of the image convolutional neural network is a set of four-dimensional tensors of input depth, width, height, and output depth [20]. Therefore, gradient processing in our algorithm is carried out in the form of matrix. SGD-MS algorithm makes different treatment for each parameter in the matrix and judges whether *V* term or *D* term should be separated according to the radius of curvature. Since SGD-MS algorithm only uses a small batch of data in each optimization step, the gradient direction of two different batches is greatly different at the beginning of training, and high-frequency noise is generated in the optimization process. The future gradient is calculated based on derivative and is very sensitive to noise, so the separation rules are shown as follows: when the curvature radius is large, it switches to *V* mode. When the radius of curvature is small, the accumulated gradient produces local optimal oscillation, and *D* mode is switches. When the situation is between the above two, *S* mode is adopted.

### 3.2. SGD-MS Optimizer Algorithm

The SGD-MS algorithm uses the internal relationship between the curvature radius of the objective function and the gradient descent of the optimizer. According to the gradient descent convergence, SGD-MS algorithm switches to *V*, *D*, and *S* modes adaptively. Pseudocode is provided in Algorithm 1.

In our algorithm, *V* and *D* represent the past gradient and the future gradient, respectively, and a moving average attenuation is added. It considers the historical value of the current step and calculates the weighted average value of these values. In the calculation result of function *ge*, the position where the curvature radius is less than *ρ*1 and the momentum term matrix is set to 0. Similarly, the calculation result of function le sets the position of curvature radius greater than *ρ*2 in the future gradient-moving average term matrix to 0. The formulas of *ge* and le are as follows:

(9) $$\begin{array}{c} ge\left(V,\rho 1\right)=0.5{}^{*}V{}^{*}mask1+0.5{}^{*}V, \end{array}$$

(10) $$\begin{array}{c} \text{le}\left(D,\rho 2\right)=0.5{}^{*}D{}^{*}mask2+0.5{}^{*}D, \end{array}$$

where *mask*1 and *mask*2 represent the values in the matrix of the corresponding position after adaptive clamping of the radius of curvature, which are set to 1 within the threshold range and −1 without the range.

The algorithm first initializes the past and future gradients to 0, then clamps the past and future gradients with the curvatures *ρ*1 and *ρ*2 of the descending gradient, and finally smooths the clamped parameters by moving average attenuation. The process is iterated successively. The specific calculation method has been shown in the pseudocode.

**Figure 2:**
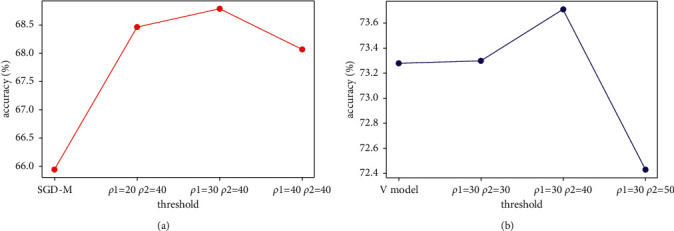
Comparison of experimental results with different values of *ρ*1 and *ρ*2. (a) *ρ*1 changes *ρ*2=40. (b) *ρ*2 changes *ρ*1=30.

In order to obtain the widely applicable values of *ρ*1 and *ρ*2, we have done several groups of comparative experiments on different datasets and networks. Taking the training results of CIFAR100 on Resnet as an example, the comparison results are shown in Table 1. The learning rate decreases from 0.1, 0.01, and 0.001 and training for 10, 100, and 150 epochs, respectively. It can be found from the table that when *ρ*1=30 and *ρ*2=40, higher accuracy can be obtained. We change the values of *ρ*1 and *ρ*2, respectively, and the comparison results are shown in Figure 2.

Through the theoretical support in the previous section and the experimental results in Figure 2, it is concluded that the smaller the *ρ*1, the more similar performance of the optimizer with SGD-M. With the increase of *ρ*1, the performance of SGD-MS will gradually deteriorate after reaching the peak. The smaller *ρ*2 is, the closer it is to *V* mode. Similar to *ρ*1, with the increase of *ρ*2, the optimizer performance first improves and then decreases. Fine tuning will be made according to this feature. In this paper, we set *ρ*1 and *ρ*2 as 30 and 40, respectively.

Equations (9) and (10) correspond to the operation of momentum term separation and future gradient-moving average term separation. In momentum separation, the position of curvature radius less than the threshold value in parameter matrix *V* is treated as 0. It is equivalent to removing momentum term, while the position with curvature radius greater than the threshold value remains unchanged, which will alleviate the local optimal oscillation problem and improve the dynamic performance. The separation of gradient moving average terms disposes the position of curvature radius greater than threshold value in matrix *D* in the same way to reduce the interference of outliers, and the position less than threshold remains unchanged, which greatly weakens the fluctuation problem.

### 3.3. Accelerated Version of SGD-MS Optimizer (SGD-MA)

Momentum is a simple and widely used technique that allows gradient-based optimizers to speed up in the direction of low curvature. Its performance mainly depends on the damping coefficient *α*. Larger values of *α* may lead to greater acceleration, but are prone to oscillations and instability; therefore, smaller values are usually used. In this paper, the idea of aggregated momentum [14] is used, which is a kind of momentum variable combining multiple velocity vectors and different *α* parameters. It has the advantages of small *α* value and large *α* value: large *α* value allows significant increase of velocity along the direction of low curvature, while small value restrains oscillation and stabilizes the algorithm. It is related to the principle of physical resonance: when the system is driven at a specific frequency, resonance will occur. Passive damping will solve this problem by using different materials with unique resonance frequency. However, the damping coefficient of this method is a fixed value set in advance and is not able to change automatically according to the gradient descent process. It has poor adaptability to different super-parameter settings in the iterative process.

We improve the gradient descent algorithm after separating partial momentum terms and future gradient-moving average term. The damping coefficient changes dynamically according to the gradient descent process. In each optimization step, the momentum and future gradient are updated, and then, the final momentum and future gradient used to update the parameters is obtained by averaging. The iteration process is written as follows:

(11) $$\begin{array}{c} \left\{\begin{array}{c} V_{t+1}=\alpha V_{t}+\frac{\partial L_{t}}{\partial \theta_{t}},\\ D_{t+1}=\alpha D_{t}+\left(1-\alpha \right)\left(\frac{\partial L_{t}}{\partial \theta_{t}}-\frac{\partial L_{t-1}}{\partial \theta_{t-1}}\right),\\ \theta_{t+1}=\theta_{t}-\alpha r\left(V_{t+1}+D_{t+1}\right)-\frac{r\;\partial L_{t}}{\partial \theta_{t}}. \end{array}\right. \end{array}$$

Equation (11) is interpreted as the weighted average value of gradient update and momentum update. Compared with each iteration, the damping coefficient is understood as a change form so that the damping coefficient is updated dynamically. *α* is initialized to 0.9, and the damping coefficient updating equation is as follows:

(12) $$\begin{array}{c} \left\{\begin{array}{c} \alpha_{t+1}r\left(V_{t+1}+D_{t+1}\right)+\frac{r\;\partial L_{t}}{\partial \theta_{t}}=\alpha_{t}r\left(\alpha_{t}V_{t}+\frac{\partial L_{t}}{\partial \theta_{t}}+\alpha_{t}D_{t}+\left(1-\alpha_{t}\right)\left(\frac{\partial L_{t}}{\partial \theta_{t}}-\frac{\partial L_{t-1}}{\partial \theta_{t-1}}\right)\right)+\frac{r\;\partial L_{t}}{\partial \theta_{t}},\\ \alpha_{t+1}=\frac{\alpha_{t}^{2}\left(V_{t}+D_{t}\right)+2\alpha_{t}\left(\partial L_{t}/\partial \theta_{t}\right)-\alpha_{t}^{2}\left(\partial L_{t-1}/\partial \theta_{t-1}\right)}{\left(V_{t+1}+D_{t+1}\right)}. \end{array}\right. \end{array}$$

**Figure 3:**
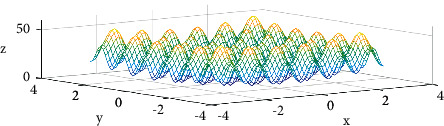
Rastrigin function.

**Figure 4:**
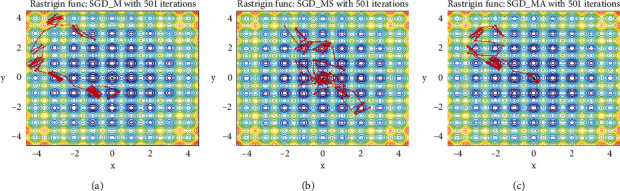
Optimizer iteration process. (a) SGD-M. (b) SGD-MS. (c) SGD-MA.

Rastrigin function [21] is a nonconvex function, which has a global minimum at (0.0, 0.0). As shown in Figure 3, since the function has a large search space and a large number of local minima, it is quite difficult to find the minimum value of the function.  Figure 4 shows how SGD-M, SGD-MS, and SGD-MA perform 501 optimization steps on the Rastrigin function in the form of top view. The minimum loss values of the three algorithms are 0.95, 0.37, and 0.33, respectively.

It is seen from Figure 4 that SGD-M has the problem of local optimal oscillation, and it is difficult to reach the global minimum after 501 steps. SGD-MS algorithm can effectively escape from the local optimum to find the global optimum. SGD-MA's iterative path is faster, which further speeds up the process of reaching the global optimum on the basis of SGD-MS algorithm. SGD-MA not only accelerates the convergence at low curvature but also achieves stable convergence at high curvature by setting adaptive damping coefficient.

## 4. Experiments

In this paper, MNIST handwritten numeral datasets are trained by using LeNet network to show the advantages of the SGD-MS optimizer and the SGD-MA optimizer. Then, the PASCAL VOC dataset is tested on Resnet models with different depths, highlighting that the improved algorithm effectively solved the problem of local optimal oscillation. Finally, CIFAR datasets are tested on Resnet [22] and Densenet [23]. All super parameters (the damping coefficient is 0.9, and the learning rate is specified in the experiment part) in the SGD-MS optimizer are set to be the same as SGD-M, which proves that the SGD-MS optimizer is superior to SGD-M in precision and has faster convergence speed. The SGD-MA optimizer further improves the convergence speed of the SGD-MS optimizer. The computer configuration used in the experiment is Intel Core i7-9700u, 32 GB RAM, and GPU is GeForce RTX 2080Ti.

### 4.1. MNIST Dataset Experimental Results

**Figure 5:**
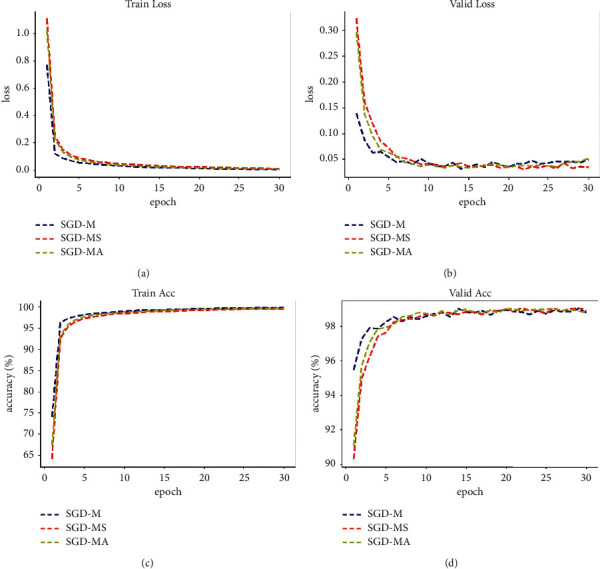
Comparison of experimental results of MNIST dataset. (a) Train loss. (b) Valid loss. (c) Train accuracy. (d) Valid accuracy.

The MNIST dataset [24] is 0 to 9 handwritten data containing 60000 training samples and 10000 test samples. The image is a grayscale image with 28 × 28 pixels. In the experiment, the SGD-MS optimizer and the SGD-MA optimizer are used to train LeNet network on MNIST dataset and compared with the SGD-M optimizer. The training is carried out in small batch with 128 images for 30 epochs, and the learning rate is set to 0.01. The comparison results are shown in Figure 5. From the figure, it is evident that the accuracy and loss values of the optimizer training set basically overlap. Compared with SGD-M, the SGD-MS optimizer significantly reduces the loss and accuracy oscillation in the test set, and the average accuracy rate exceeds SGD-M after the 8th epoch. From the loss figure, SGD-M algorithm has overfitting phenomenon after the 15th epoch, and the accuracy rate remains basically unchanged in the subsequent iterations, while SGD-MS algorithm performs well. SGD-MA algorithm improves the convergence speed based on SGD-MS, and the accuracy continues to rise steadily. It is concluded that the SGD-MS optimizer not only has small loss and high precision but also has high generalization ability, and the SGD-MA optimizer accelerates the convergence speed while maintaining the performance of SGD-MS.

### 4.2. PASCAL VOC Dataset Experimental Results

The PASCAL VOC dataset is the standard image dataset and standard evaluation system provided by the PASCAL VOC challenge for detecting algorithms and learning performance. The dataset has good image quality and complete annotation, including a total of 20 classes. With the continuous development of computer vision technology, small- and medium-sized datasets are becoming more and more popular in the research field. In order to obtain a better image classification effect, the existing research usually adopts the pretraining method, which can not only save the calculation time but also improve the recognition effect. Therefore, it is necessary to use this method to verify the improved optimizer. In order to obtain more image features, the pretraining parameters from the Imagenet dataset were applied to the Resnet model with the depth of 18, 34, and 50, respectively, in this experiment. During the training process, the PASCAL VOC dataset was trained by means of parameter fine tuning. The backbone learning rate is set to 0.001, the FC learning rate to 0.05, the batch size to 32, and 15 epochs to be iterated. The final test accuracy and loss values trained by the SGD-M, SGD-MS, and SGD-MA optimizer are shown in Table 2, where the maximum accuracy and minimum loss are shown in bold.

**Figure 6:**
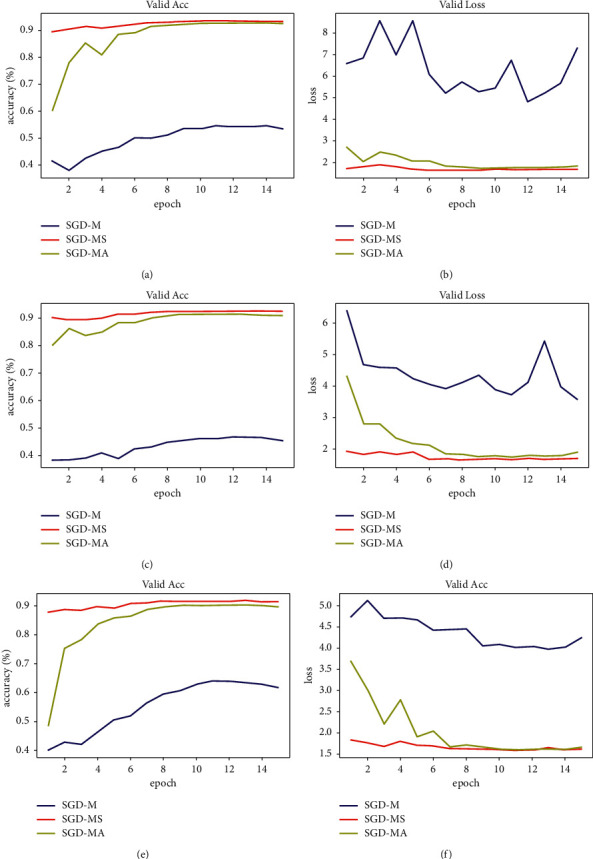
Comparison results of different algorithms for PASCAL VOC dataset under the Resnet model. (a) Resnet18 accuracy. (b) Resnet18 loss. (c) Resnet34 accuracy. (d) Resnet34 loss. (e) Resnet50 accuracy. (f) Resnet50 loss.

It can be clearly seen from Table 2 that the performance of the proposed improved algorithm is better than SGD-M. Under the same parameter setting, SGD-M shows the phenomenon of training failure, but the two proposed improved optimizers still perform well. We can make a specific analysis of this phenomenon through Figure 6.

As can be seen from Figure 6, during the training of Resnet models with different depths, the SGD-M optimizer has serious local optimal oscillation phenomenon in the gradient descending process, which makes it difficult to improve the accuracy and ultimately leads to the training failure. Two improved algorithms, SGD-MS and SGD-MA, have overcome this problem and achieved good results. The pretraining mode was adopted in this experiment, which can be understood as saving a large period of pretraining time, which made the acceleration function of SGD-MA not fully reflected. The next experiment provided supplementary demonstration.

### 4.3. CIFAR Dataset Experimental Results

In order to test the generality of our method to deeper model and larger dataset, we choose two widely used datasets CIFAR10 and CIFAR100 and model Resnet and Densenet. CIFAR10 and CIFAR100 datasets [25] are composed of 60000 RGB color images with a resolution of 32 × 32, including 10 and 100 classes, respectively, and are divided into 50000 training images and 10000 test images. In the experiment, Resnet and Densenet models are used to compare SGD-M, SGD-MS, and SGD-MA algorithms on CIFAR10 and CIFAR100, respectively, to highlight the importance of adaptive mode switching. The initial learning rate of the experiment was 0.1, and it decreased to 0.01 at the 10th epoch and to 0.001 at the 100th epoch, totally 150 epochs. The results are summarized in Table 3 (iteration stage is represented by the model/epoch number). The second column in the table shows the depth of these networks. The third, fourth, and fifth rows show the average accuracy of cifar10 after 10 epochs, 100 epochs, and 150 epochs. The sixth, seventh, and eighth rows list the average accuracy rates of cifar100 after 10 epochs, 100 epochs, and 150 epochs, and the optimal value is expressed in bold.

**Figure 7:**
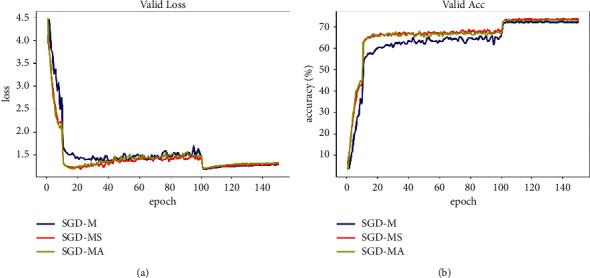
Comparison results of different algorithms for CIFAR 100 dataset under the Resnet model. (a) Loss. (b) Accuracy.

The data in Table 3 can reflect that, for the two models used on the two CIFAR datasets, SGD-MS and SGD-MA optimizers are all higher than or equal to SGD-M except for the experimental results of the 150th epoch in Densenet, which are slightly lower than SGD-M. The result may be related to the Densenet dense connection structure, which can mitigate the gradient disappearance. When the learning rate is small, the gradient decline decreases, and the influence of network structure is dominant, and the effect of network is offset with the effect of the improved optimizer to reduce the oscillation. Although the threshold and initial parameters of the two improved optimizers are tested with default values, on the whole, they achieve a fairly good effect, which further proves the wide applicability of the threshold value and saves a lot of adjustment time in practice.  Figure 7 shows the experimental results of CIFAR100 for 150 epochs in an optional experimental test set iteration on Resnet. All models achieve nearly perfect accuracy on the training set, so we will not explain it. It can be seen from the figure that compared with SGD-M, SGD-MS algorithm always performs well and has significantly less fluctuation in the process of gradient decline, which effectively alleviates the problem of local optimal oscillation, and the loss can be reduced to a lower level and the accuracy can reach a higher level. It is clear that the SGD-MS algorithm is superior to SGD-M in comparison. Though the validation set accuracy of the SGD-MA optimizer in some datasets' training models is slightly lower than that of SGD-MS, it is still much higher than SGD-MA, and it can converge on the training target quickly, especially in large learning rate iteration. Although SGD-MA could not compete with SGD-MS in test accuracy, it showed better training performance and faster convergence in higher learning rate.

## 5. Conclusions

SGD-MS and SGD-MA optimizers proposed in this paper are good substitutes for the SGD-M optimizer. SGD-MS uses the internal relationship between curvature radius and gradient descent and takes radius of curvature as threshold to realize automatic switching of *V*, *D*, and *S* modes, which improves the local optimal oscillation, shortens the convergence time, and increases training accuracy. The SGD-MA algorithm introduces the adaptive damping coefficient on this basis, which improves the convergence speed while maintaining the good verification accuracy of the SGD-MS optimizer. As the introduction of additional parameters, each iteration of this algorithm will take a little more time than SGD-M algorithm. However, due to the weakening of local optimum oscillation, the system converges faster, the total iteration time is shortened, the learning process is accelerated, and the labour and time cost are greatly saved. In future work, we will investigate how to associate SGD-MS and SGD-MA optimizers with adaptive learning rates and maintain good generalization performance.

## Figures and Tables

**Algorithm 1:**
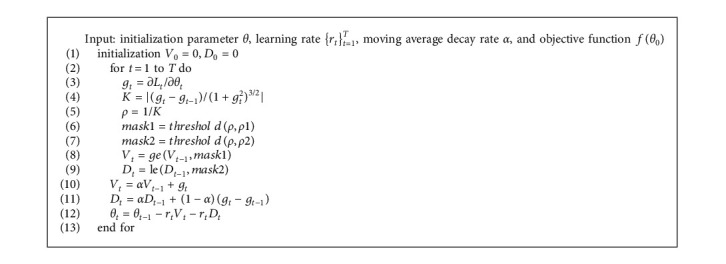
SGD-MS.

**Table 1:** Comparison results of different curvature radii as thresholds.

Epochs	10	100	150
SGD-M	36.37	65.94	72.62
*ρ*1=20*ρ*2=40	43.11	68.47	73.11
*ρ*1=30*ρ*2=40	42.89	68.79	73.71
*ρ*1=40*ρ*2=40	46.09	68.07	72.42
*ρ*1=30*ρ*2=30	47.15	68.03	73.3
*ρ*1=30*ρ*2=50	43.3	67.58	72.43
*ρ*1=30*ρ*2=*∗*	46.63	68.29	73.28

**Table 2:** Test results.

Algorithm (SGD-M/MS/MA)	Resnet18	Resnet34	Resnet50
Accuracy	64.00/**91.70**/90.00	46.60/**92.60**/91.50	54.60/**93.30**/92.60
Loss	3.58/**1.68**/1.79	4.80/**1.67**/1.76	3.98/**1.59**/1.61

**Table 3:** Results' summary.

Model/epoch number	Algorithm (SGD-)	Resnet	Densenet
Depth	*∗*	116	100
CIFAR10/10	M/MS/MA	70.79/**77.21**/73.15	79.60/81.87/**87.23**
CIFAR10/100	M/MS/MA	90.70/**91.10**/90.89	91.16/91.32/**91.37**
CIFAR10/150	M/MS/MA	**93.67**/**93.67**/93.44	**94.00**/93.84/93.81
CIFAR100/10	M/MS/MA	36.37/42.89/**44.87**	46.68/**51.70**/49.99
CIFAR100/100	M/MS/MA	65.94/**68.79**/67.82	68.88/**69.01**/68.46
CIFAR100/150	M/MS/MA	72.62/**73.71**/73.31	**74.87**/71.85/71.98

## Data Availability

All data included in this study are available from the corresponding author upon request.
